# Regulation of iron homeostasis by the p53-ISCU pathway

**DOI:** 10.1038/srep16497

**Published:** 2015-11-12

**Authors:** Yuki Funauchi, Chizu Tanikawa, Paulisally Hau Yi Lo, Jinichi Mori, Yataro Daigo, Atsushi Takano, Yohei Miyagi, Atsushi Okawa, Yusuke Nakamura, Koichi Matsuda

**Affiliations:** 1 Laboratory of Molecular Medicine, Human Genome Center, Institute of Medical Science, The University of Tokyo, Tokyo, Japan; 2 Center for Antibody and Vaccine Therapy, Research Hospital, Institute of Medical Science, The University of Tokyo, Tokyo 108-8639, Japan; 3 Department of Medical Oncology and Cancer Center, Shiga University of Medical Science, Otsu 520-2192, Japan; 4 Molecular Pathology and Genetics Division, Kanagawa Cancer Center, Yokohama 241-0815, Japan; 5 Department of Orthopedic Surgery, Tokyo Medical and Dental University, Tokyo, Japan; 6 Departments of Medicine and Surgery and Center for Personalized Therapeutics, The University of Chicago, USA

## Abstract

Accumulation of iron in tissues increases the risk of cancer, but iron regulatory mechanisms in cancer tissues are largely unknown. Here, we report that p53 regulates iron metabolism through the transcriptional regulation of *ISCU* (iron-sulfur cluster assembly enzyme), which encodes a scaffold protein that plays a critical role in Fe-S cluster biogenesis. p53 activation induced ISCU expression through binding to an intronic p53-binding site. Knockdown of ISCU enhanced the binding of iron regulatory protein 1 (IRP1), a cytosolic Fe-S protein, to an iron-responsive element in the 5′ UTR of *ferritin heavy polypeptide 1* (*FTH1*) mRNA and subsequently reduced the translation of FTH1, a major iron storage protein. In addition, in response to DNA damage, p53 induced FTH1 and suppressed transferrin receptor, which regulates iron entry into cells. HCT116 *p53*^+/+^ cells were resistant to iron accumulation, but HCT116 *p53*^−/−^ cells accumulated intracellular iron after DNA damage. Moreover, excess dietary iron caused significant elevation of serum iron levels in *p53*^−/−^ mice. ISCU expression was decreased in the majority of human liver cancer tissues, and its reduced expression was significantly associated with *p53* mutation. Our finding revealed a novel role of the p53-ISCU pathway in the maintenance of iron homeostasis in hepatocellular carcinogenesis.

The tumour suppressor gene *p53* is mutated in more than half of all human cancers^1^. Although recent cancer genomics studies have identified a number of genes that are mutated in human cancer, none of them have a higher frequency of somatic mutation than the *p53* gene^2^. Because over 90% of missense mutations observed in the p53 gene are clustered within its DNA-binding domain^3^, sequence-specific transcriptional regulation is considered to be one of its critical functions in suppressing human tumours. In response to various types of cellular stress, activated p53 regulates many target genes, including those involved in cell cycle arrest, apoptosis, DNA repair, and cellular senescence^4^. By means of cDNA microarray analyses, we previously isolated a number of p53 target genes, including p53RDL1, XEDAR, and PADI4^5^,^6^,^7^,^8^,^9^. However, the p53 signalling pathway has not yet been completely elucidated.

Recently, accumulating evidence has suggested significant roles of iron metabolism in the development of cancer^10^. Iron is an essential mineral for both normal and cancer cells, and iron depletion has deleterious effects on mammalian systems, such as oxygen transport, electron transfer, and catalysis^11^. Moreover, excess iron also causes various problems, including DNA damage due to the generation of hydroxyl radicals such as reactive oxygen species (ROS)^12^. Epidemiological analyses have indicated a link between iron storage in the body and the risk of cancer. A high intake of dietary iron is associated with an increased risk for several cancers, such as colorectal^13^ and liver cancer^14^. Iron accumulation is frequently observed in liver cancer tissues^15^; patients with hereditary haemochromatosis, who exhibit high iron deposition particularly in liver parenchymal cells, were shown to have a 20- to 200-fold higher risk for liver cancer^16^. However, the detailed mechanisms of iron dysregulation in cancer tissues remain to be elucidated. Here, we report a novel mechanism by which p53 regulates iron homeostasis through the induction of its transcriptional target.

## Results

### Identification of ISCU as a p53-inducible gene

**Figure 1:**
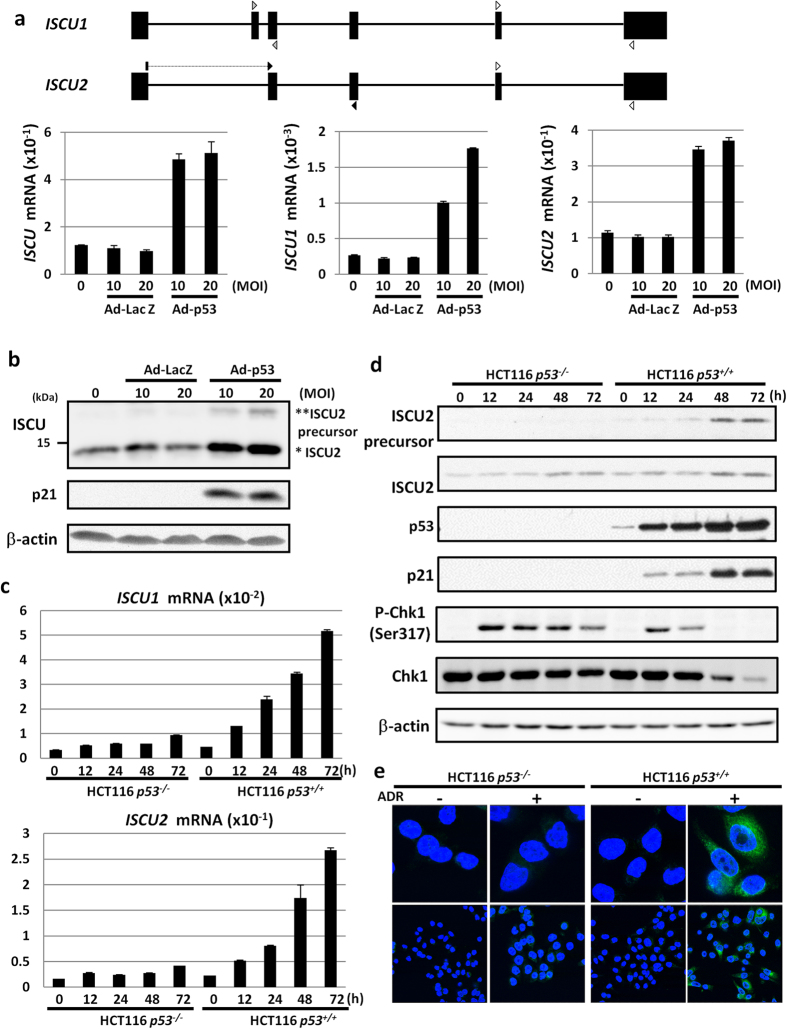
Induction of ISCU by p53. (**a**) Genomic structure of *ISCU* and locations of the primers for quantitative real-time PCR (qPCR) specific to each isoform (upper). Black boxes indicate the location and relative size of the 6 exons. Arrows indicated primer sets for *ISCU1* (gray arrow), *ISCU2* (black arrow), and common *ISCU* (white arrow). qPCR analysis of *ISCU* in U373MG (p53 mutant) cells infected with adenovirus expressing p53 (Ad-p53) or LacZ (Ad-LacZ) at a multiplicity of infection (MOI) of 10 or 20 (lower). *ACTB* was used to normalize the expression levels. Error bars represent the S.D. (n = 3). (**b**) U373MG cells were infected with Ad-p53 or Ad-LacZ at an MOI of 10 or 20. Thirty-six hours after treatment, whole cell extracts were subjected to immunoblotting with an anti-ISCU, anti-p21, or anti-β-actin antibody. *ISCU2, **ISCU2 precursor. (**c**,**d**) HCT116 *p53*^−/−^ or HCT116 *p53*^+/+^ cells were treated with adriamycin (ADR). At the indicated time after treatment, total RNA or whole cell extracts were subjected to qPCR (**c**) or immunoblotting (**d**) using an anti-ISCU, anti-p53, anti-p21, anti-P-Chk1 (Ser317), anti-Chk1, or anti-β-actin antibody. (**e**) HCT116 *p53*^−/−^ or HCT116 *p53*^+/+^ cells were treated with ADR. Thirty-six hours after treatment, cells were fixed and stained with an anti-ISCU antibody (Alexa Fluor 488; green). DAPI was used to visualize the nuclei (blue). Upper, magnified images.

To identify novel p53 target genes, we conducted a cDNA microarray analysis using mRNAs isolated from p53-mutant U373MG glioblastoma cells that were infected with adenovirus designed to express wild-type p53 (Ad-p53) or LacZ (Ad-LacZ)^6^. Through this screening system, we identified more than 60 genes that were upregulated by the induction of ectopic wild-type p53 expression. To verify the microarray analysis, we performed quantitative real-time PCR (qPCR) analyses and confirmed that the expression of *ISCU* (iron-sulfur cluster assembly enzyme) was remarkably induced by the introduction of p53 in a dose-dependent manner but not by that of control LacZ (Fig. 1a). ISCU is a scaffold protein that is crucial for iron-sulfur (Fe-S) cluster biogenesis. Because Fe-S proteins that contain an Fe-S cluster exhibit diverse biochemical properties in the mitochondrial respiratory chain and enzyme activity^17^, the Fe-S cluster is an essential cofactor for all living organisms. Defects in ISCU function cause ISCU myopathy, which is characterized by exercise-induced lactic acidosis and muscle weakness^17^. However, the role of ISCU in human carcinogenesis has not been reported previously.

*ISCU* has two major isoforms, *ISCU1* and *ISCU2*, both of which are involved in the generation of the Fe-S cluster^17^. Hence, we examined the expression of *ISCU1* and *ISCU2* using specific primer sets and found that both isoforms were induced by p53 (Fig. 1a). Because *ISCU2* expression is nearly one hundred-fold higher than that of *ISCU1*, ISCU2 is considered to be the major isoform induced by p53. ISCU2 protein is rapidly processed to a mature form of approximately 14 kDa^18^. In agreement with the qPCR results, the amounts of precursor and mature ISCU2 protein were increased by p53 in a dose-dependent manner (Fig. 1b and Supplementary Fig. S1a). p21, a major p53-inducible cell cycle regulator, was used as a positive control for p53 activation^19^. Induction of *ISCU1*/*2* by Ad-p53 was also observed in p53-deficient H1299 cells (Supplementary Fig. S1b).

### Induction of ISCU by DNA damage

**Figure 2:**
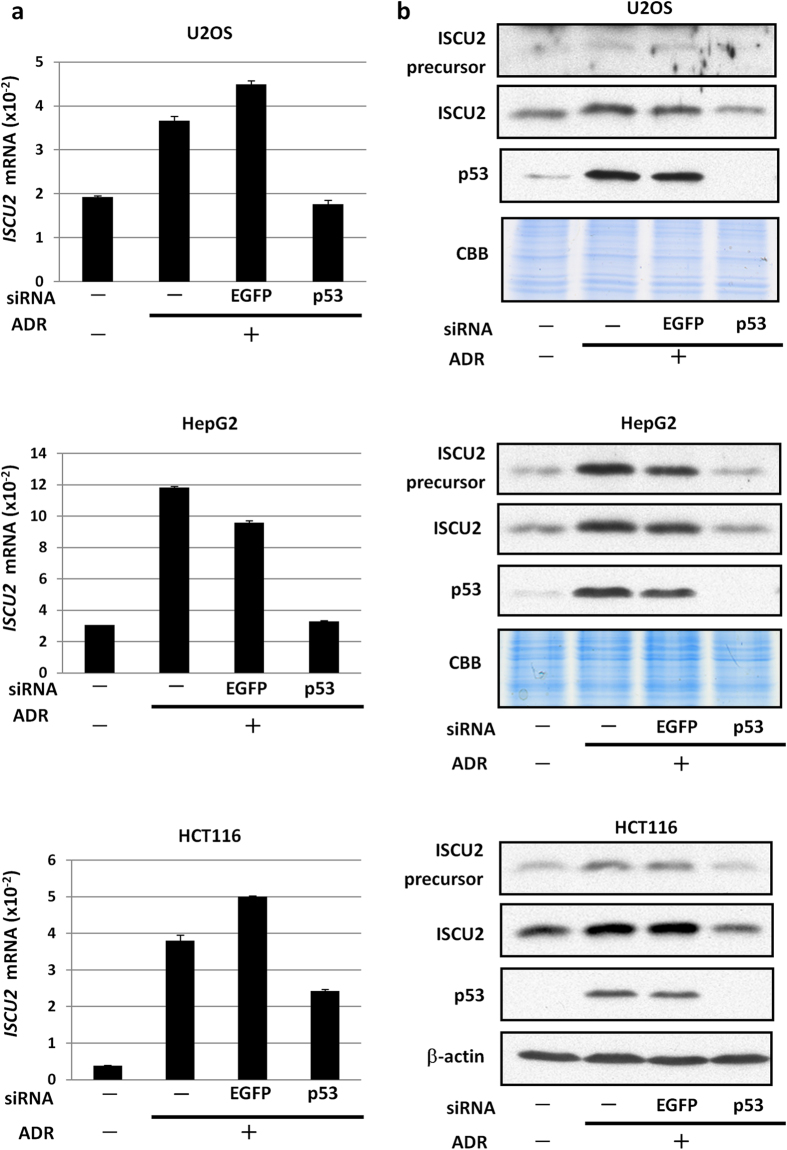
Induction of ISCU by DNA damage. (**a**) Twenty-four hours after transfection of each siRNA, U2OS (p53 wild-type), HepG2 (p53 wild-type) and HCT116 (p53 wild-type) cells were treated with adriamycin (ADR). After 36 h of treatment, cells were collected, and quantitative real-time PCR (qPCR) analyses were performed. ACTB was used to normalize the expression levels. Error bars represent the S.D. (n = 3). (**b**) Twenty-four hours after transfection of each siRNA, U2OS (p53 wild-type), HepG2 (p53 wild-type) and HCT116 (p53 wild-type) cells were treated with adriamycin. After 36 h, cell extracts were subjected to western blot analysis. siRNA against EGFP was used as the control. CBB staining and β-actin are shown as loading controls.

To examine the regulation of *ISCU* by endogenous p53, we investigated the expression of *ISCU* using HCT116 *p53*^+/+^ and HCT116 *p53*^−/−^ cells treated with adriamycin (ADR). Although *ISCU* mRNA expression was slightly increased in HCT116 *p53*^−/−^ cells, *ISCU* expression was more than five-fold higher in HCT116 *p53*^+/+^ cells after ADR treatment (Fig. 1c). We also demonstrated the induction of ISCU2 protein expression by ADR treatment in HCT116 *p53*^+/+^ cells (Fig. 1d). Phosphorylation of Chk1 at ser 317, a mediator of DNA damage signalling, was observed 12 h after ADR-treatment in both cells. Subsequent immunocytochemical analysis revealed that ISCU was expressed in the cytoplasm of HCT116 *p53*^+/+^ cells in response to ADR but was present at very low levels in HCT116 *p53*^−/−^ cells (Fig. 1e). ISCU2 is synthesized as a precursor which is located in the cytosol and then migrates to the mitochondria after mitochondrial target sequence cleavage, while ISCU1 which lacks target sequence localizes at cytoplasm [2]. Based on the result of qPCR and western blot analysis, accumulation of ISCU protein in the cytoplasm of ADR-treated HCT116 *p53*^+/+^ cells would reflect the induction of ISCU2 precursor protein. The expression of *ISCU2* mRNA and ISCU2 protein was also induced in U2OS, HepG2, and HCT116 cells after ADR treatment, but siRNA against p53 markedly inhibited ISCU2 expression (Fig. 2a,b), further supporting the p53-dependent induction of ISCU in response to DNA damage.

### ISCU is a direct target of p53

**Figure 3:**
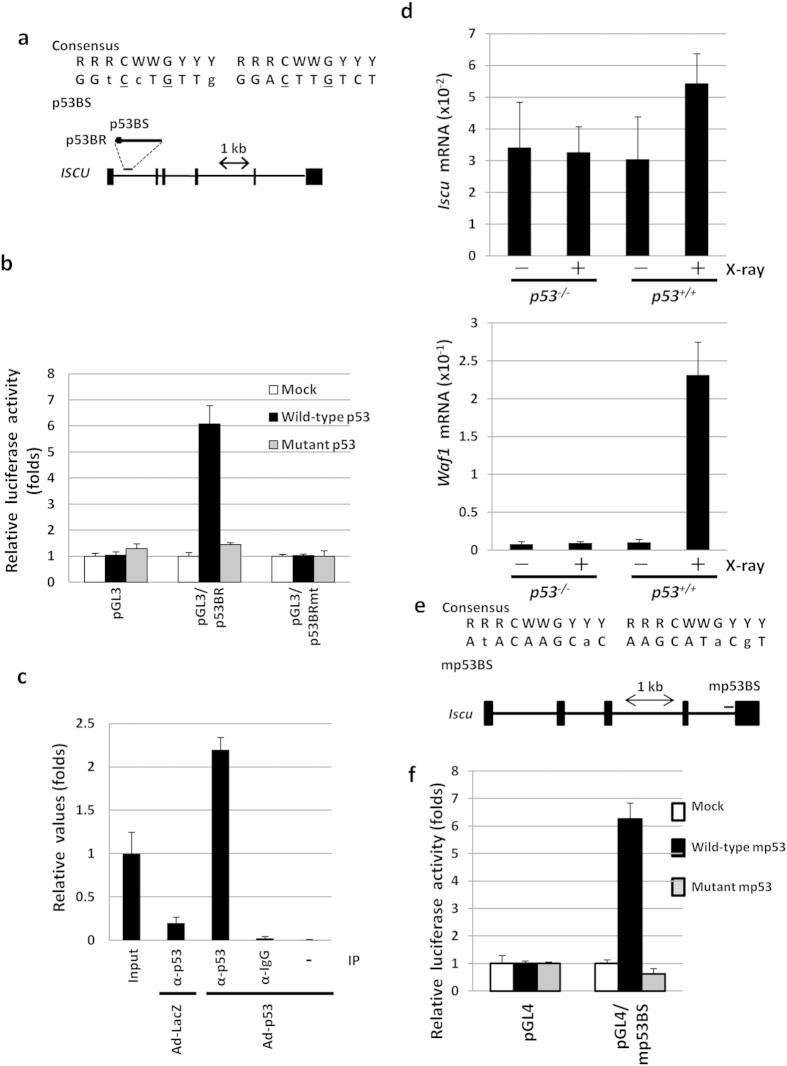
ISCU is a direct target of p53. (**a**) (Upper) Comparison of p53BS to the consensus p53-binding sequence. R, purine; W, A or T; Y, pyrimidine. Identical nucleotides to the consensus sequence are written in capital letters. The underlined cytosine and guanine were substituted for thymine to examine the specificity of the p53-binding site. (Lower) Genomic structure of the *ISCU* gene. The box indicates the location of the potential p53-binding sequence (p53BS) in the p53-binding region (p53BR). (**b**) Luciferase assay of p53BR with or without mutation of the p53BS in U373MG cells. Luciferase activity is indicated relative to the activity of the mock vector; error bars represent the S.D. (n = 3). The mutant p53 plasmid harbours a missense mutation (R175H) in p53. (**c**) ChIP was performed using U373MG cells that were infected with Ad-p53 (lanes 1 and 3-5) or Ad-LacZ (lane 2). DNA-protein complexes were immunoprecipitated with an anti-p53 antibody (lanes 2 and 3) followed by qPCR analysis. Input chromatin represents a small portion (1%) of the sonicated chromatin before immunoprecipitation (lane 1). Immunoprecipitates with normal mouse IgG (lane 4) or in the absence of antibody (lane 5) were used as negative controls. Columns, mean; error bars, S.D. (n = 3). (**d**) *p53*^+/+^ or *p53*^−/−^ mice received 10 Gy of X-ray irradiation at 6 weeks of age. Twenty-four hours after treatment, thymus tissues were collected, and a qPCR analysis of *Iscu* and *Waf1* expression was performed. Non-irradiated *p53*^+/+^ or *p53*^−/−^ mice were used as controls. Actb was used to normalize the expression levels. (**e**) Potential p53-binding sequence (mp53BS) in the 4^th^ intron of the mouse *Iscu* gene. (Upper) Comparison of p53BS to the consensus p53-binding sequence. R, purine; W, A or T; Y, pyrimidine. Identical nucleotides to the consensus sequence are written in capital letters. (Lower) Genomic structure of the mouse *Iscu* gene. Bar indicates the location of the mp53BS. (**f**) Luciferase assays were performed using mp53BS oligonucleotides subcloned into pGL4.24. Luciferase activity is indicated relative to the activity of the mock vector; error bars represent the S.D. (n = 3).

To examine whether *ISCU* is a direct target of p53, we surveyed the genomic sequence of the *ISCU* gene located on chromosome 12q24.1 and found a putative p53-binding sequence (p53BS) within the first intron (Fig. 3a). The p53BS showed an 85% (17/20) match to the consensus p53-binding sequence^20^ (Fig. 3a). We then subcloned a 300-bp DNA fragment (p53BR) containing this p53BS into the pGL3 promoter plasmid (pGL3/p53BR) and evaluated the p53-dependent transcriptional activity using a reporter assay. Luciferase activity was strongly enhanced by cotransfection of pGL3/p53BR and wild-type p53 but not by that of pGL3/p53BR and a mutant form of p53 (Fig. 3b). In addition, base substitutions within the p53BS (pGL3/p53BRmt) completely abolished the enhancement of the luciferase activity (Fig. 3b). To further verify whether p53 could bind to this DNA segment, we performed a chromatin immunoprecipitation (ChIP) assay using U373MG cells that were infected with either Ad-p53 or Ad-LacZ. qPCR analysis of the immunoprecipitated DNA indicated that the p53 protein bound to the genomic fragment that included the p53BS (Fig. 3c). Taken together, these findings implied that p53 directly regulated *ISCU* expression through binding to the p53BS in the first intron.

Then, we analysed the induction of *Iscu in vivo*. RNA was purified from the thymus of *p53*^+/+^ and *p53*^−/−^ mice 24 h after 10 Gy of X-ray irradiation. The qPCR analysis demonstrated that *Iscu* mRNA was increased after DNA damage in *p53*^+/+^ mice but not in *p53*^−/−^ mice (Fig. 3d). To investigate whether Iscu is a target of mouse p53, we screened for the p53BS in the mouse *Iscu* locus and found a putative p53BS (mp53BS) in the fourth intron (Fig. 3e). We subcloned the mp53BS into the pGL4.24 plasmid and conducted a reporter assay. Luciferase activity was strongly enhanced by cotransfection with wild-type p53 but not by that of a mutant form of p53 (Fig. 3f). These findings indicated that both human ISCU and mouse Iscu are targets of p53.

### Regulation of IRP1 activity and FTH1 expression by the p53-ISCU pathway

Activated p53 inhibits cell growth by inducing the expression of its target genes that are involved in apoptotic cell death or cell cycle arrest. To analyse the function of ISCU, we examined whether the ectopic expression of ISCU affects tumour cell growth. We performed colony formation assays using three cancer cell lines (HCT116, colorectal cancer; U373MG, glioblastoma; H1299, lung cancer) transfected with plasmids expressing mock or ISCU2 and found that ISCU2 did not remarkably affect the growth of these cancer cells (Supplementary Fig. S2).

**Figure 4:**
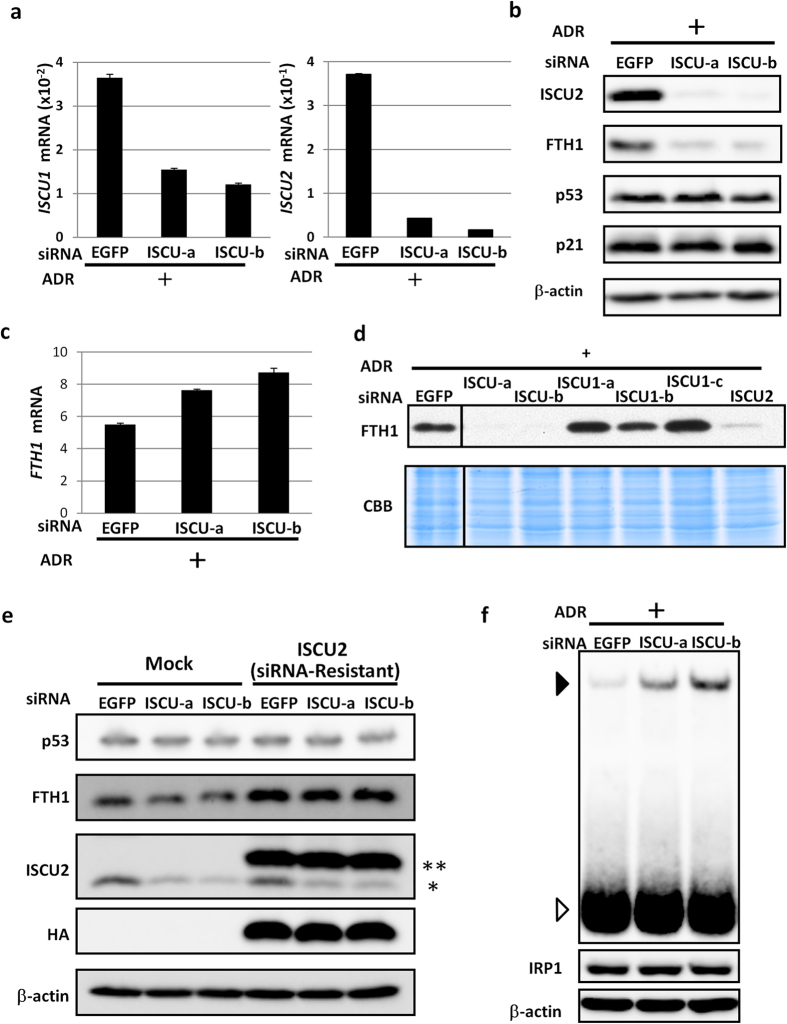
Regulation of FTH1 expression by ISCU. (**a**) Twenty-four hours after transfection of each siRNA, HCT116 cells were treated with ADR. After 36 h, cells were collected, and qPCR analysis was performed. siRNA against EGFP was used as a control. *ACTB* was used to normalize the expression levels. Error bars represent the S.D. (n = 3). (**b**) Twenty-four hours after transfection of each siRNA, HCT116 cells were treated with ADR. After 36 h, cell extracts were subjected to western blot analysis. siRNA against EGFP was used as a control. β-actin is shown as a loading control. (**c**) Twenty-four hours after transfection of each siRNA, HCT116 cells were treated with ADR. After 36 h, cells were collected, and qPCR analysis was performed. siRNA against *EGFP* was used as a control. *ACTB* was used to normalize the expression levels. Error bars represent the S.D. (n = 3). (**d**) Twenty-four hours after transfection of each siRNA, HCT116 cells were treated with 2 μg/ml of ADR. After 36 h, cells were harvested for western blot assay. CBB staining is shown as a loading control. ISCU1-a, ISCU1-b, ISCU1-c, or ISCU2 indicate siRNA specific for ISCU1 or ISCU2, respectively. (**e**) Twenty-four hours after transfection of the mock or ISCU2 expression vector, HCT116 cells were treated with siRNA. After 24 h, HCT116 cells were incubated with 2 μg/ml of ADR. Cells were collected for western blot assay 36 h after ADR treatment. β-actin is shown as a loading control. **Exogenous ISCU2 (HA-tagged ISCU2). *Endogenous ISCU2. (**f**) RNA-EMSA using a biotin-labelled probe containing the IRE in the 5′ UTR of *FTH1* mRNA. Twenty-four hours after transfection of each siRNA, HCT116 cells were treated with ADR. After 36 h, cytosolic cellular fractions were incubated with probe for 30 min, and electrophoresis mobility shift assays were performed (upper). The open arrowhead indicates free probe, and the closed arrowhead indicates the protein-RNA complex. Cytosolic fractions were also subjected to western blot analysis using an anti-IRP1 or anti-β-actin antibody (lower).

ISCU acts as a scaffold protein for Fe-S cluster biogenesis. Iron regulatory protein 1 (IRP1, also known as ACO1) is an Fe-S protein that controls the expression of various iron regulatory proteins^21^,^22^. The Fe-S cluster inhibits the interaction between IRP1 and iron regulatory elements (IRE) in transcripts of iron regulatory genes, such as *ferritin heavy polypeptide 1* (*FTH1*)^23^,^24^. Therefore, we investigated the possible role of the p53-ISCU pathway in iron homeostasis. We generated two small interfering RNAs (siRNAs) designed to simultaneously suppress both ISCU isoforms and confirmed the suppression of *ISCU* mRNA expression in HCT116 cells treated with ADR (Fig. 4a). We then analysed the expression of FTH1 after ISCU knockdown and observed a marked reduction in FTH1 protein levels (Fig. 4b), but *FTH1* mRNA was not reduced by ISCU knockdown (Fig. 4c).

To further elucidate the role of ISCU1/2 in FTH1 regulation, we designed siRNAs specific for ISCU1 and ISCU2. HCT116 cells treated with siISCU2 exhibited a marked reduction in FTH1 expression, whereas siISCU1 did not affect FTH1 levels (Fig. 4d). In addition, HCT116 cells sequentially transfected with a plasmid expressing an siRNA-resistant form of ISCU2 and siISCU showed higher expression of FTH1 (Fig. 4e). These findings indicated that ISCU2 plays a major role in the regulation of FTH1 expression.

*FTH1* mRNA contains an IRE in its 5′ untranslated region (UTR). When IRP1 interacts with IRE, it inhibits mRNA translation and consequently suppresses FTH1 protein expression^25^. Hence, our findings suggested that ISCU might regulate the IRP1-IRE interaction through Fe-S cluster biogenesis and subsequently enhance the translation of FTH1. To further investigate our hypothesis, we conducted RNA electrophoretic mobility shift assays (EMSAs). HCT116 cells were transfected with either siISCU or siEGFP and then treated with ADR. Cytosolic fractions of the cells were incubated with a biotinylated RNA fragment containing an IRE derived from the 5′ UTR of *FTH1*. The labelled products were subjected to gel electrophoresis. We found increased IRE-protein complex formation after ISCU knockdown without any effect on IRP1 expression (Fig. 4f). Competition with non-labelled IRE probe and IRP1 knockdown markedly reduced the intensity of the shifted band (Supplementary Fig S3a–c), indicating the specificity of IRP1-IRE interaction. These results suggested that the p53-ISCU pathway positively modulated FTH1 protein levels through the IRP1-IRE regulatory system.

### Regulation of intracellular iron by p53

**Figure 5:**
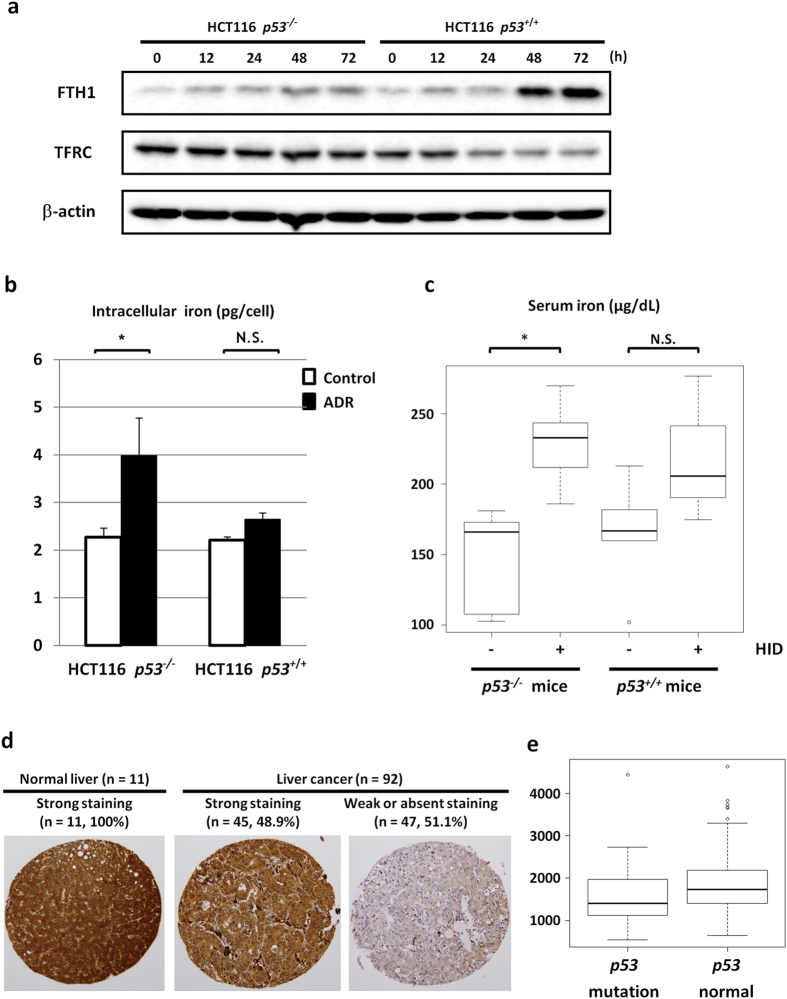
Regulation of iron homeostasis by p53. (**a**) Western blot analyses of *FTH1* and *TFRC* in HCT116 *p53*^−/−^ or HCT116 *p53*^+/+^ cells harvested at the indicated times after ADR treatment (2 μg/ml for 2 h). β-actin was used to normalize the expression levels. (**b**) HCT116 *p53*^+/+^ or HCT116 *p53*^−/−^ cells were treated with ADR. After 36 h, cells were collected to measure intracellular iron levels. Error bars represent the S.D. (n = 3). *P < 0.05 by Welch’s t-test. (**c**) *p53*^+/+^ or *p53*^−/−^ mice at 6 weeks of age were fed a high-iron diet (HID) for 3 weeks. At 9 weeks of age, serum iron levels were examined. *p53*^+/+^ or *p53*^−/−^ mice (9 weeks of age) fed a normal diet were used as controls. *p53*^+/+^ + normal diet (n = 5); *p53*^+/+^ + HID (n = 3); *p53*^−/−^ + normal diet (n = 5); *p53*^−/−^ + HID (n = 8). Error bars represent the S.D. *P < 0.05 by Welch’s t-test. (**d**) Immunohistochemical staining of ISCU in human liver cancer tissues (n = 92) and normal liver tissues (n = 11) (magnification × 100). (**e**) Expression of *ISCU* in hepatocellular carcinoma tissues with mutant p53 (n = 60) or wild-type p53 (n = 311) was quantified by RSEM^58^. All data were retrieved at cBioPortal (http://www.cbioportal.org/public-portal/index.do).

Because an IRE is present in various iron regulatory genes, we further investigated the effect of p53 activation on these proteins. ADR treatment induced the protein expression of FTH1 only in HCT116 *p53*^+/+^ cells (Fig. 5a). Conversely, the protein expression of transferrin receptor (TFRC) was downregulated in HCT116 *p53*^+/+^ cells after ADR treatment but was not markedly affected in HCT116 *p53*^−/−^ cells (Fig. 5a). Similarly, TFRC expression was higher in sip53-treated HCT116 cells than in control cells at both the RNA and protein levels (Supplementary Fig. S4a and S4b). Unlike FTH1, *TFRC* mRNA contains five IREs in its 3′ UTR^23^, and the interaction between IRP1 and the IRE in the 3′ UTR stabilizes the mRNA and consequently increases the protein levels^25^. Hence, p53 would be expected to increase FTH1 protein and decrease TFRC protein through regulation of the IRP1-IRE interaction. To test this hypothesis, we conducted RNA EMSA using a biotinylated RNA fragment containing an IRE derived from the 3′ UTR of *TFRC*. Similar to the result with *FTH1*, IRE-protein complex formation was increased in cells treated with siISCU (Supplementary Fig. S4c). These findings indicated that the p53-ISCU pathway also regulated TFRC expression by inhibiting IRP1 binding to the IRE in the 3′ UTR of *TFRC*.

Because TFRC has a direct effect on intracellular iron levels by mediating the uptake of transferrin-bound iron^10^, we further investigated intracellular iron levels in HCT116 *p53*^+/+^ or HCT116 *p53*^−/−^ cells via the ferrozine method^26^ after DNA damage. Interestingly, DNA damage significantly increased intracellular iron levels in HCT116 *p53*^−/−^ cells, but HCT116 *p53*^+/+^ cells were resistant to iron elevation after DNA damage (Fig. 5b). In addition, ISCU depletion increased intracellular iron levels in ADR-treated HCT116 cells (Supplementary Fig. S5a). These findings suggested that the p53-ISCU pathway plays an important role in the regulation of iron homeostasis.

We also analyzed gene expression profile of ADR-treated HCT116 *p53*^+/+^ and *p53*^−/−^ cells and found that *transferrin* was induced by DNA damage in both HCT116 *p53*^+/+^ and *p53*^−/−^ cells (Supplementary Fig. S5b). Because transferrin-bound iron is transported into cells through transferrin receptor, *transferrin* induction could explain increased iron level in HCT116 *p53*^−/−^ cells after DNA damage. Our findings indicated that FTH1 expression was slightly increased in HCT116 *p53*^−/−^ cells after ADR treatment (Fig. 5a). Fth1 was shown to be regulated by Fbxl5-mediated IRP2 degradation when iron is abundant^27^. Moreover, Fth1 is induced by transcription factor Nrf2 (nuclear factor E2 p45-related factor 2) which responds to diverse oxidative and electrophilic environmental stresses^28^. Therefore, Fth1 would be induced by several mechanisms regardless of p53 status.

### Regulation of iron metabolism by p53 *in vivo*

To investigate whether p53 regulates iron homeostasis *in vivo*, we examined the effect of dietary iron overload using *p53*^+/+^ and *p53*^−/−^ mice. At 6 weeks of age, *p53*^+/+^ and *p53*^−/−^ mice were fed a high-iron diet (HID) or a normal diet for 3 weeks. *Iscu* mRNA levels were slightly increased in the liver of *p53*^+/+^ mice fed the HID compared with *p53*^−/−^ mice (Supplementary Fig. S6a). In addition, Tfrc expression was significantly decreased in *p53*^+/+^ mice (Supplementary Fig. S6b,c). These findings suggested that p53 regulated iron regulatory proteins *in vivo*.

We also analysed serum iron levels. Basal serum iron levels were not different between the *p53*^+/+^ and *p53*^−/−^ mice fed the normal diet. Although the HID did not significantly affect serum iron levels in the *p53*^+/+^ mice, it significantly increased serum iron levels in the *p53*^−/−^ mice (Fig. 5c).

### Role of ISCU in hepatocellular carcinoma

In agreement with previous reports^29^, qPCR analyses of 38 normal human tissues revealed strong expression of ISCU in multiple tissues, including normal liver (Supplementary Fig. S7). Because excess iron was reported to increase the risk of liver cancer^14^, we investigated the role of the p53-ISCU pathway in hepatocellular carcinogenesis. We analysed ISCU expression in 11 normal human liver tissues and 92 human liver cancer tissues by tissue microarray analysis. Although ISCU was strongly expressed in all of the normal tissues, its expression was decreased (weak or absent) in more than half of the liver cancer tissues (51.1%, 47/92, Fig. 5d), suggesting a possible tumour suppressive function of ISCU in liver. ISCU expression in liver cancer tissues was inversely associated with p53 staining, although this relationship was not statistically significant (P = 0.107, Supplementary Table S2). Because cancer tissues harbouring mutant p53 generally exhibit an accumulation of p53 protein^30^,^31^, this inverse association corresponded with our findings that ISCU is a direct target of p53. We also investigated the association between *ISCU* expression and p53 mutation status in 371 hepatocellular carcinoma tissues (The Cancer Genome Atlas (TCGA) Data portal; https://tcga-data.nci.nih.gov/tcga/). Interestingly, *ISCU* expression was significantly higher in hepatocellular carcinoma tissues with wild-type p53 compared to those with mutant p53 (Fig. 5e, 1843.3 ± 618.1 vs 1567.7 ± 647.7, P = 2.9 × 10^−4^)^32^, further supporting the *in vivo* regulation of ISCU by p53.

## Discussion

Recently, accumulating evidence has suggested a relationship between metal ions and cancer. Iron accumulation results in DNA damage via ROS production and subsequently activates the cMYC^33^ and WNT^34^ pathways. Similarly, zinc deficiency or excess copper, chrome, nickel, cadmium, or arsenic was shown to promote cancer development by enhancing ROS production^35^ or epigenetic disorder^36^. However, the regulatory mechanisms involving metal ions in cancer tissues largely remain to be elucidated. Although the role of p53 in the regulation of FTH1 and TFRC expression through the modulation of IRP1 activity was previously reported^37^, the molecular mechanism whereby p53 modulates IRP1 activity was not elucidated. Furthermore, the effects of p53 on intracellular or serum iron levels were not investigated previously.

**Figure 6:**
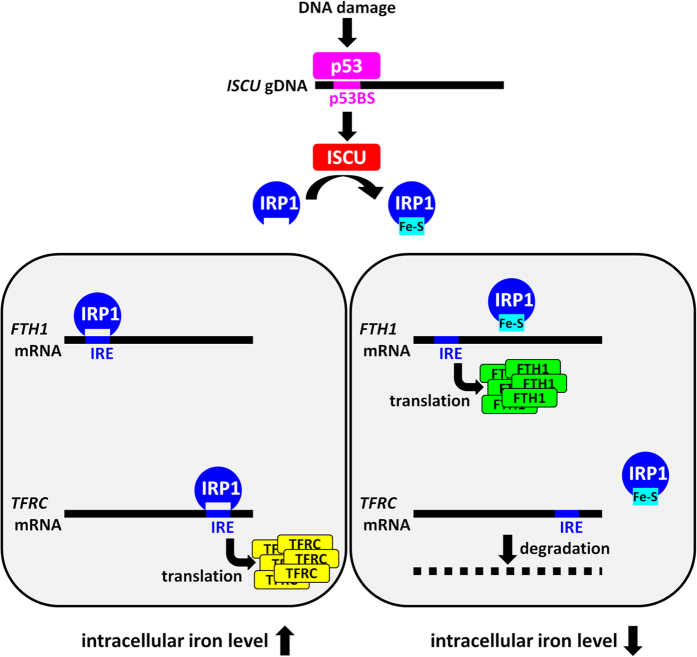
A model for the role of p53-ISCU pathway in iron regulation. Following DNA damage, p53 induces expression of ISCU, a scaffold protein that plays a critical role in Fe-S cluster biogenesis. Fe-S cluster inhibits binding of IRP1 to an iron-responsive element (IRE) in the 5′ UTR of *FTH1* and 3′ UTR of *transferrin receptor* (*TFRC*), and subsequently increases the translation of FTH1 and reduces *TFRC* mRNA. FTH1 functions as a major iron storage protein and TFRC regulates iron entry into cells. Therefore, p53-ISCU pathway would play an important role in the maintenance of iron homeostasis.

Our findings revealed that the p53-ISCU pathway controlled FTH1 and TFRC expression through an IRP1-IRE regulatory system (Fig. 6). TFRC facilitates iron entry, while FTH1 stores and detoxifies excess intracellular iron^38^. Therefore, the p53-ISCU pathway would have a protective effect against iron overload. To the best of our knowledge, this is the first report suggesting the role of p53 in the maintenance of metal ion homeostasis *in vivo*. However, systemic iron homeostasis is regulated by multiple mechanisms including intestinal iron uptake, iron exporter ferroportin, and iron consumption in the body. Because most of our experiments were conducted using cell lines, further analysis is necessary to elucidate the role of p53-ISCU pathway in the regulation of systemic iron.

As is the case in many types of cancer, iron accumulation is frequently observed in hepatocellular carcinoma^15^. Iron chelation or bloodletting can improve the prognosis of a liver cancer patient as well as curtail liver injury^39^. In the genomic analyses of liver cancers, the most frequently mutated gene is *p53*^40^. Furthermore, ISCU expression was reduced in half of the liver cancer tissues (51.1%, 47/92) in our analysis. ISCU was shown to be a target of miR-210 that was frequently increased in cancer tissues^41^, and ISCU was significantly downregulated in ovarian cancers^42^ and medulloblastoma^43^. Taken together, the data suggest that ISCU is a potential tumour suppressor that is inactivated through various mechanisms, such as p53 inactivation and miR-210 upregulation.

In this study, we analysed the role of ISCU in iron homeostasis and hepatocellular carcinogenesis; however, ISCU is ubiquitously expressed in most human organs^29^,^44^. Excess iron is associated with various cancers, including breast, kidney, colon, pancreas, bladder, oesophagus, and stomach cancer^10^, and low *ISCU* expression is associated with poor prognosis in breast cancer patients^45^. Moreover, Fe-S proteins exhibit diverse biochemical properties, such as DNA helicase activity^46^, aconitase activity, and electron transport chain activity in mitochondria^47^. Therefore, loss of Fe-S cluster biogenesis might impair DNA repair machinery by inhibiting DNA helicase activity or reduce TCA cycle activity and subsequently stimulate aerobic glycolysis (also known as the Warburg effect)^48^, a typical metabolic change that occurs in cancer cells.

IRP1 functions as aconitase in cytoplasm when it binds Fe-S cluster. Our result indicated that DNA damage activates p53-ISCU pathway which would lead to Fe-S cluster biosynthesis and the induction of aconitase activity. Therefore we evaluated aconitase activity in HCT116 cells, but we did not observe significant induction of aconitase activity by ADR treatment (data not shown). This result could be partially explained by the negative feedback loop which is triggered by the dissociation of IRP1 from IRE within *FTH1* and *TFRC* mRNA and subsequent reduction of free intracellular iron level. In addition, aconitase activity was controlled by various mechanisms including oxidative stress which was regulated by several p53 downstream targets^49^,^50^. Therefore, further analysis is essential to elucidate the association of p53-ISCU pathway with cytosolic aconitase activity in DNA damage response. We also analyzed expression of other Fe-S proteins by using the result of cDNA microarray analysis conducted in HCT116 *p53*^+/+^ and *p53*^−/−^ cells and found the p53-dependent induction of *RSAD2* and *FDX1L* genes (Supplementary Fig. 8). *RSAD2* encodes an interferon inducible iron-sulfur cluster binding-protein which plays a major role in anti viral activity and inhibits a wide range of DNA and RNA viruses such as human cytomegalovirus, hepatitis C virus, and west Nile virus^51^. The *FDX1L* gene which encodes protein with a 2Fe-2S ferredoxin-type domain is essential for heme A and Fe/S protein biosynthesis and associated with mitochondrial muscle myopathy^52^. Thus, p53 might regulate various physiological processes by controlling Fe-S cluster proteins. Although further analyses are essential to fully elucidate the role of the p53-ISCU pathway in carcinogenesis, our findings provide the first step in clarifying the role of p53 in the maintenance of iron homeostasis.

## Materials and Methods

### cDNA microarray

cDNA microarray analysis was performed as described previously^6^. Briefly, U373MG cells were infected with viral solutions and incubated at 37 °C until harvest. Polyadenylated RNA was isolated from U373MG cells using standard protocols. Each RNA sample was labelled and hybridized to a microarray consisting of 36,864 cDNA fragments. The dataset is available in the GEO database (http://www.ncbi.nlm.nih.gov/geo/) as GSE14953.

### Plasmid construction

The entire coding sequence of ISCU2 cDNA was amplified by PCR using KOD-Plus DNA polymerase (Toyobo, Osaka, Japan) and inserted into the *Eco*RI and *Xho*I sites of the pCAGGS vector. The construct was confirmed by DNA sequence analysis. To create an siRNA-resistant ISCU2 expression vector, we inserted point mutations in the siRNA target sequences (siISCU-a and siISCU-b) by site-directed mutagenesis without changing the ISCU2 amino acid sequence. Primers used for amplification and mutagenesis are shown in Supplementary Table S1.

### Cell culture and transfection

The U373MG (glioblastoma), H1299 (lung carcinoma), HCT116 (colorectal adenocarcinoma), U2OS (osteosarcoma), and HepG2 (hepatocellular carcinoma) human cancer cell lines were purchased from American Type Culture Collection. HCT116 (*p53*^+/+^ and *p53*^−/−^) cell lines were gifts from B. Vogelstein (Johns Hopkins University, Baltimore, MD, USA). Cells were transfected with plasmids using FuGENE6 (Roche, Basel, Switzerland), Lipofectamine LTX or Lipofectamine 2000 (Invitrogen, Carlsbad, CA). Replication-deficient recombinant viruses, Ad-p53 or Ad-LacZ, expressing p53 or LacZ were generated and purified as described previously^53^. U373MG or H1299 cells were infected with viral solutions at various multiplicity of infection (MOI) values and incubated at 37 °C until harvest. Small interfering RNA (siRNA) oligonucleotides, commercially synthesized by Sigma Genosys (Hokkaido, Japan), were transfected using Lipofectamine RNAiMAX reagent (Invitrogen, Carlsbad, CA, USA). The siRNA oligonucleotide sequences are shown in Supplementary Table S1.

### DNA damaging treatment

To elicit genotoxic stress, cells were continuously incubated with 2 μg/ml of adriamycin (ADR) for 2 h. Mice were exposed to X-ray irradiation using an X-ray irradiation system (MBR-1520R-3, Hitachi).

### Quantitative real-time PCR (qPCR)

Total RNA was isolated from cells or thymus tissues using RNeasy Plus Spin Column Kits or RNeasy Plus Universal Mini Kits (Qiagen, Valencia, CA, USA) according to the manufacturer’s instructions. Poly A^+^ RNA or total RNA from normal human tissues was purchased from Clontech (Mountain View, CA, USA). Complementary DNAs were synthesized using the SuperScript Preamplification System (Invitrogen, Carlsbad, CA, USA). qPCR was conducted with SYBR Green Master Mix on a LightCycler 480 (Roche, Basel, Switzerland). Primer sequences are shown in Supplementary Table S1.

### Western blot analysis and immunocytochemistry

To prepare whole cell extracts, cells were collected and lysed in chilled radio-immunoprecipitation assay (RIPA) buffer [50 mmol/L Tris-HCl (pH 8.0), 150 mmol/L NaCl, 0.1% SDS, 0.5% sodium deoxycholate, and 1% NP40] for 30 min on ice and centrifuged at 16,000 × *g* for 15 min. Samples were subjected to SDS-PAGE and immunoblotting using standard procedures. Immunocytochemistry was performed as described previously^6^.

### Antibodies

Anti-β-actin monoclonal antibody (A5441) was purchased from Sigma-Aldrich (St. Louis, MO, USA). Anti-TFRC (transferrin receptor) monoclonal antibody (sc-65882) and anti-Chk1 monoclonal antibody (sc-8408) was purchased from Santa Cruz Biotechnology (Santa Cruz, CA, USA). Anti-ISCU polyclonal antibody (14812-1-AP) was purchased from Proteintech (Chicago, IL, USA). Anti-IRP1 monoclonal antibody (ab126595) was purchased from Abcam (Cambridge, UK). Anti-p21 monoclonal antibody (05-345), anti-p53 (OP43), and anti-phospho Ser 317 Chk1 polyclonal antibody (DR1025) were purchased from Merck Millipore (Darmstadt, Germany). Anti-FTH1 polyclonal antibody (#3998) was purchased from Cell Signaling (Beverly, MA, USA).

### Gene reporter assay

A DNA fragment including the potential p53-binding site (p53BS) of *ISCU* was amplified and subcloned into the pGL3-promoter (human *ISCU*) or pGL4.24 (mouse *Iscu*) vector (Promega, Madison, WI, USA). To create a mutant vector, point mutations were introduced at the 4th and 14th nucleotides (C to T mutations) and the 7th and 17th nucleotides (G to T mutations) within the consensus p53BS by site-directed mutagenesis. Reporter assays were performed using the Dual Luciferase Assay System (pGL3-promoter) or the Dual-Glo Luciferase Assay System (pGL4.24) (Promega, Madison, WI, USA) as described previously^6^. Sequences of primers for amplification and site-directed mutagenesis are shown in Supplementary Table S1.

### Chromatin immunoprecipitation (ChIP) assay

ChIP assays were performed using EZ-Magna ChIP G Chromatin Immunoprecipitation Kits (Merck Millipore, Darmstadt, Germany) following the manufacturer’s protocol. In brief, Ad-p53- or Ad-LacZ-infected U373MG cells were cross-linked with 1% formaldehyde for 10 min, washed with PBS, and lysed in nuclear lysis buffer. The lysate was then sonicated using Bioruptor UCD-200 (Cosmo Bio, Tokyo, Japan) to shear the DNA into fragments of approximately 200–1000 bp. The supernatant from 1 × 10^6^ cells was used for each immunoprecipitation with anti-p53 antibody (OP140, Merck Millipore, Darmstadt, Germany) or normal mouse IgG (sc-2025, Santa Cruz, Santa Cruz, CA, USA). Before immunoprecipitation, 1% of the supernatant was removed as “input”. Column-purified DNA was quantified by qPCR. Primer sequences are shown in Supplementary Table S1.

### RNA Electrophoretic Mobility Shift Assay (EMSA)

The IRP1-IRE interactions were analysed using LightShift Chemiluminescent RNA Electrophoretic Mobility Shift Assay Kits (Pierce Biotechnology, Rockford, IL, USA) following the manufacturer’s protocol. Biotin-labelled IRE probes corresponding to the 5′ UTR of *FTH1* mRNA (UCUUGCUUCAACAGUGUUUGAACGGAAC) and 3′ UTR of *TFRC* mRNA (AAUUAUCGGGAACAGUGUUUCCCAUAAUU) were used in this assay. Briefly, the cytosolic cellular fraction was extracted using NE-PER Nuclear and Cytoplasmic Extraction Reagents (Thermo Fisher Scientific, Waltham, MA, USA). Ten micrograms of protein from each cell extract was incubated with biotin-labelled IRE probe at room temperature for 30 min. For competition study, the cytosolic cellular fraction was incubated with labelled IRE probe and 160-fold excess of non-labelled IRE probe. The reaction products were separated by electrophoresis in 0.5 × TBE at 100 V for 45 min, transferred to a nylon membrane in 0.5 × TBE at 35 V for 45 min, UV-crosslinked at 120 mJ/cm^2^, and imaged according to manufacturer’s instructions.

### Iron measurement

Intracellular iron levels were measured using a Metallo Assay LS Ferrozine Kit (AKJ Global Technology, Chiba, JAPAN) following the manufacturer’s instructions^26^. Briefly, 5 × 10^4^ cells were lysed in 1 ml of RIPA buffer, sonicated using a Bioruptor UCD-200 (Cosmo Bio, Tokyo, Japan), and incubated in 0.1 M HCl for 30 min. After centrifuging the samples at 20,000 × *g* for 15 min, the iron levels were determined based on the ferrozine method^54^ by measuring the absorbance at 570 nm using an ARVO X3 multilabel reader (Perkin-Elmer, Waltham, MA, USA).

### High-iron diet treatment

*p53*-deficient mice were provided by RIKEN BioResource Center (Ibaraki, Japan)^55^. The high iron-containing diet was prepared by adding 2% (w/w) ferric citrate to CA-1 (containing 0.03% [w/w] ferric citrate; CLEA Japan, Tokyo, Japan)^56^. *p53*^+/+^ and *p53*^−/−^ mice at 6 weeks of age were fed the high-iron diet or the normal diet for 3 weeks. Blood was sent to Mitsubishi Chemical Medience (Tokyo, Japan) for serum iron measurements. All mice were maintained under specific pathogen-free conditions and were handled in accordance with the Guidelines for Animal Experiments of the Institute of Medical Science (University of Tokyo, Tokyo, Japan).

### Immunohistochemistry and tissue microarray

Tumour tissue microarrays were constructed using 92 formalin-fixed, paraffin-embedded primary hepatocellular carcinoma tissues from Kanagawa Cancer Center, each of which was obtained using an identical protocol to collect, fix and preserve the tissues after resection^57^. The tissue area for sampling was selected based on visual alignment with the corresponding H&E-stained section on a slide. Three or four tissue cores (diameter, 0.6 mm; depth, 3–4 mm) taken from a donor tumour block were placed into a recipient paraffin block with a tissue microarrayer (Beecher Instruments, Sun Prairie, WI, USA). A core of normal tissue was punched from each case, and 5 μm sections of the resulting microarray block were used for immunohistochemical analysis. To investigate the ISCU/p53 protein status in clinical hepatocellular carcinoma samples, rabbit anti-ISCU antibody (14812-1-AP, Proteintech, Chicago, IL, USA), mouse anti-p53 antibody (OP140, Merck Millipore, Darmstadt, Germany), or rabbit anti-ferritin heavy chain (FTH1) antibody (sc-25617, Santa Cruz, CA, USA) was added to each slide after blocking endogenous peroxidase and proteins, and the sections were incubated with ENVISION+ kit/HRP (Dako, Carpinteria, CA, USA) as a secondary antibody. Substrate chromogen was added, and the specimens were counterstained with haematoxylin. The ISCU/p53 staining was evaluated by three independent investigators. Scoring was performed on a multi-viewer light microscope (BX53, Olympus, Tokyo, Japan) at 100× magnification. Because the intensity of ISCU staining within each tumour tissue and normal liver tissue core was mostly homogeneous, ISCU positivity was assessed semiquantitatively (strong, weak or absent). To ascertain the p53 status, p53 staining was considered positive if 10% or more cells showed positive nuclear staining. Statistical analyses were performed using the StatView statistical program (SaS). We used contingency tables to analyse the relationship between ISCU expression and clinicopathologic variables (age, virus infection, and p53 or FTH1 staining) in patients with hepatocellular carcinoma. Statistical analyses were performed using Fisher’s exact test. *P*-values less than 0.05 were considered statistically significant.

### Cell proliferation assay

Colony formation assays were conducted in six-well culture plates. Cells transfected with pCAGGS/ISCU2 or mock plasmid were cultured in the presence of geneticin (0.8, 0.8, or 0.5 mg/ml for U373MG, H1299, or HCT116 cells, respectively) (Invitrogen, Carlsbad, CA, USA) for 1–2 weeks. Colonies were stained with crystal violet (Sigma, St. Louis, MO, USA) and quantified using ImageJ software.

### cDNA microarray

Gene expression analysis was performed using SurePrint G3 Human GE 8 × 60 K microarray (Agilent, Santa Clara, CA, USA) according to the manufacturer’s protocol. Briefly, HCT116 *p53*^+/+^ or HCT116 *p53*^−/−^ cells were treated with ADR and incubated at 37 °C until the time of harvest. Total RNA was isolated from the cells using standard protocols. Each RNA sample was labeled and hybridized to array slides.

## Additional Information

**How to cite this article**: Funauchi, Y. *et al*. Regulation of iron homeostasis by the p53-ISCU pathway. *Sci. Rep*. **5**, 16497; doi: 10.1038/srep16497 (2015).

## Supplementary Material

Supplementary Information
