# Supplementary material for: Regulation of iron homeostasis by the p53-ISCU pathway
Source: Sci Rep. 2015 Nov 12;5:16497. doi: 10.1038/srep16497 (PMC4642350; doi:10.1038/srep16497)
Supplement: Supplementary Information [file srep16497-s1.pdf]

Supplementary Information

Regulation of iron homeostasis by the p53-ISCU pathway

Yuki Funauchi[1,5], Chizu Tanikawa[1], Paulisally Hau Yi Lo[1], Jinichi Mori[1], Yataro Daigo[2,3], Atsushi Takano[2,3], Yohei Miyagi[4], Atsushi Okawa[5], Yusuke Nakamura[6] and Koichi Matsuda[1]*

Supplementary Figure 1

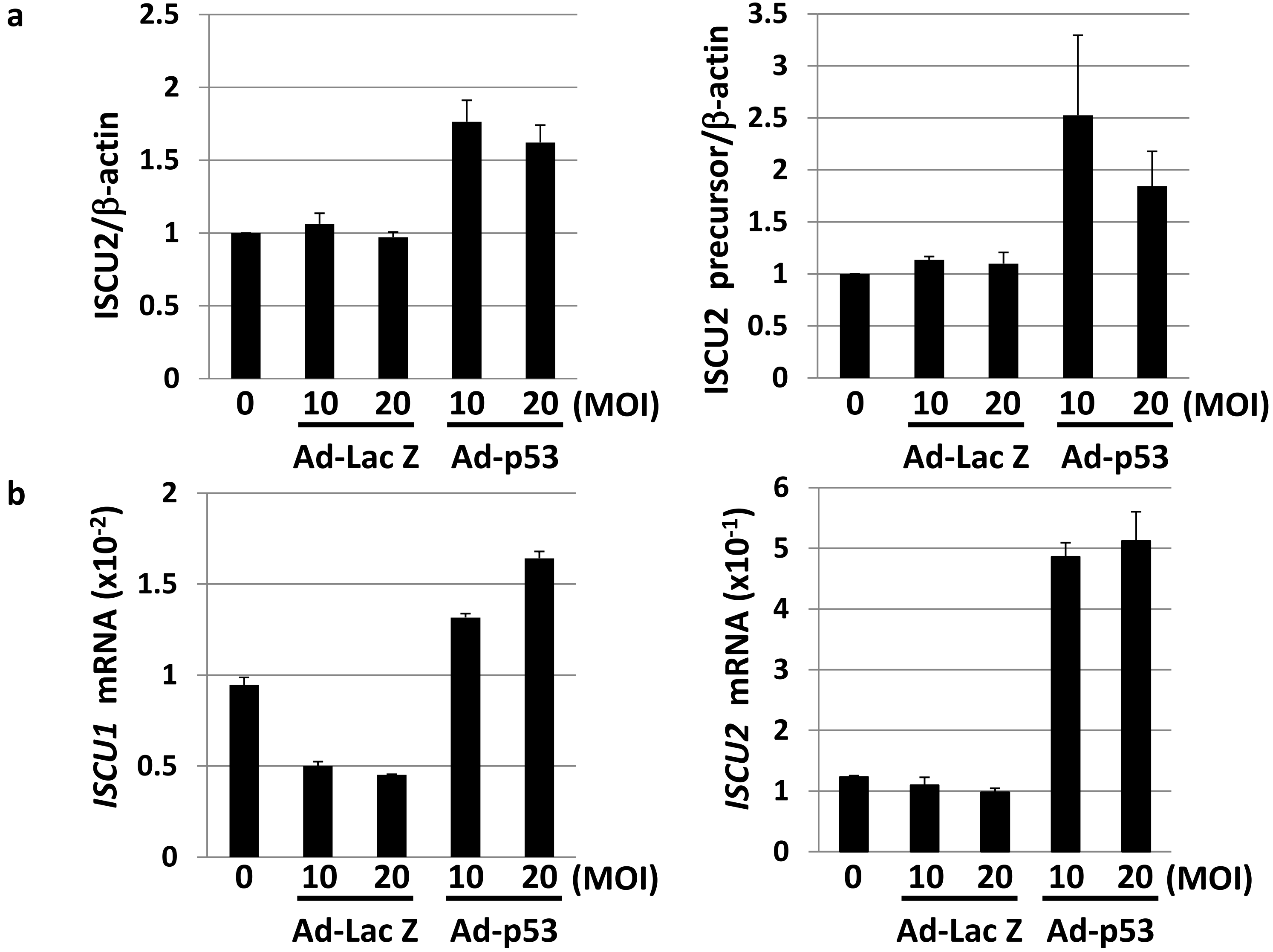

Supplementary Figure 1. **Induction of *ISCU* by p53.**

U373 (a) or H1299 (b) cells were infected with adenovirus expressing p53 (Ad-p53) or LacZ (Ad-LacZ) at a multipleultiplicity of infection (MOI) of 10 or 20. (a) Protein expressions of ISCU2 and ISCU2 precursor from Fig. 1b were quantified using Image Quant TL Analysis Toolbox (GE healthcare life science, Piscataway, NJ, USA) according to the manufacturer's instructions. β-actin was used for the normalization of expression levels. Untreated cells were taken as 1. Error bars represent S.D. (n = 3). (b) *ISCU1* and *ISCU2* expression were analyzed by quantitative PCR. *ACTB* was used for the normalization of expression levels. Error bars represent S.D. (n = 3).

Supplementary Figure 2

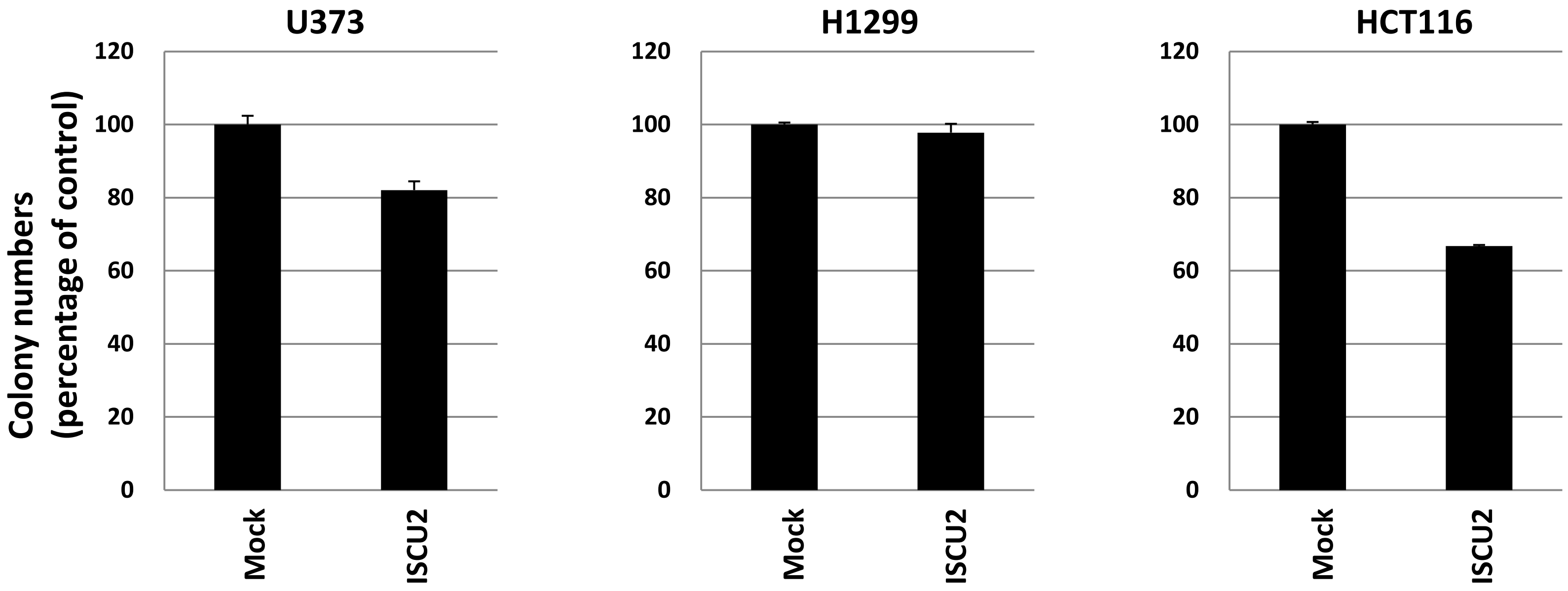

Supplementary Figure 2. Cell proliferation after overexpression of ISCU.
Colony formation assay was carried out using U373MG, H1299, and HCT116 cells. Cells were transfected with plasmids expressing mock or ISCU2, and were cultured in the presence of geneticin (0.8, 0.8, and 0.5 mg/ml for U373MG, H1299, and HCT116 cells, respectively) for 2 weeks. The number of colonies was quantified using the Image J software.

Supplementary Figure 3

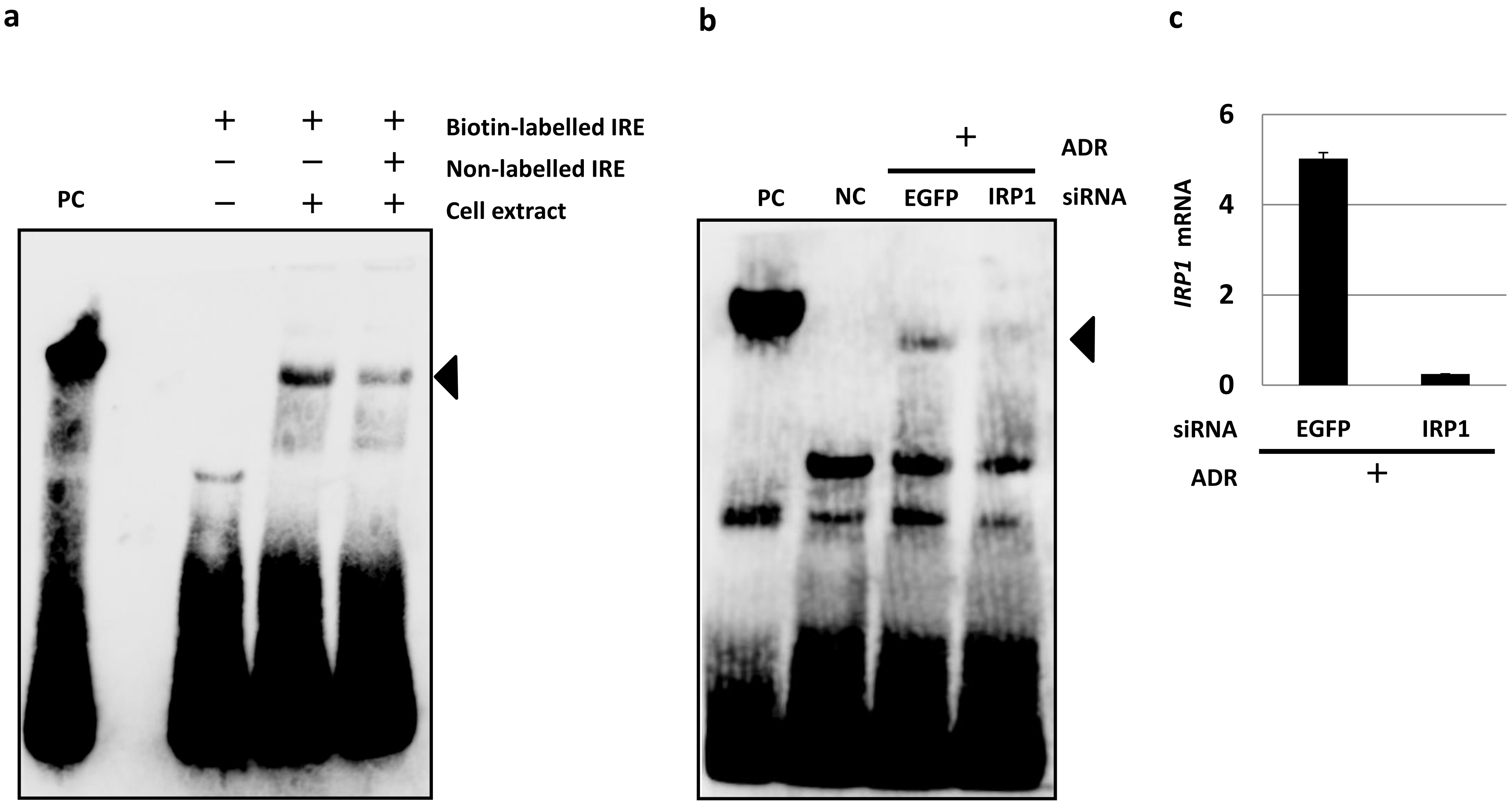

Supplementary Figure 3. RNA-EMSA using biotin-labelled or non-labelled probe containing IRE in the 5' UTR of *FTH1* mRNA.
(a) HCT116 cells were treated with ADR. After 36 h, cytosolic cellular fractions were incubated with each probe for 30min, and RNA-EMSA was performed. (b) Twenty-four hours after transfection of each siRNA, HCT116 cells were treated with ADR. After 36 h, cytosolic cellular fractions were incubated with probe for 30min, and RNA-EMSA was performed. The arrowhead indicates protein-RNA complex. The biotin-labelled probe was incubated with cytosolic liver extract (PC) or without protein extract (NC). (c) At 24 h after transfection of each siRNA, HCT116 cells were treated with adriamycin (ADR). At 36 h after treatment, qPCR analysis were performed. EGFP was used as control. *ACTB* was used for the normalization of expression levels. Error bars represent S.D. (n = 3)

Supplementary Figure 4

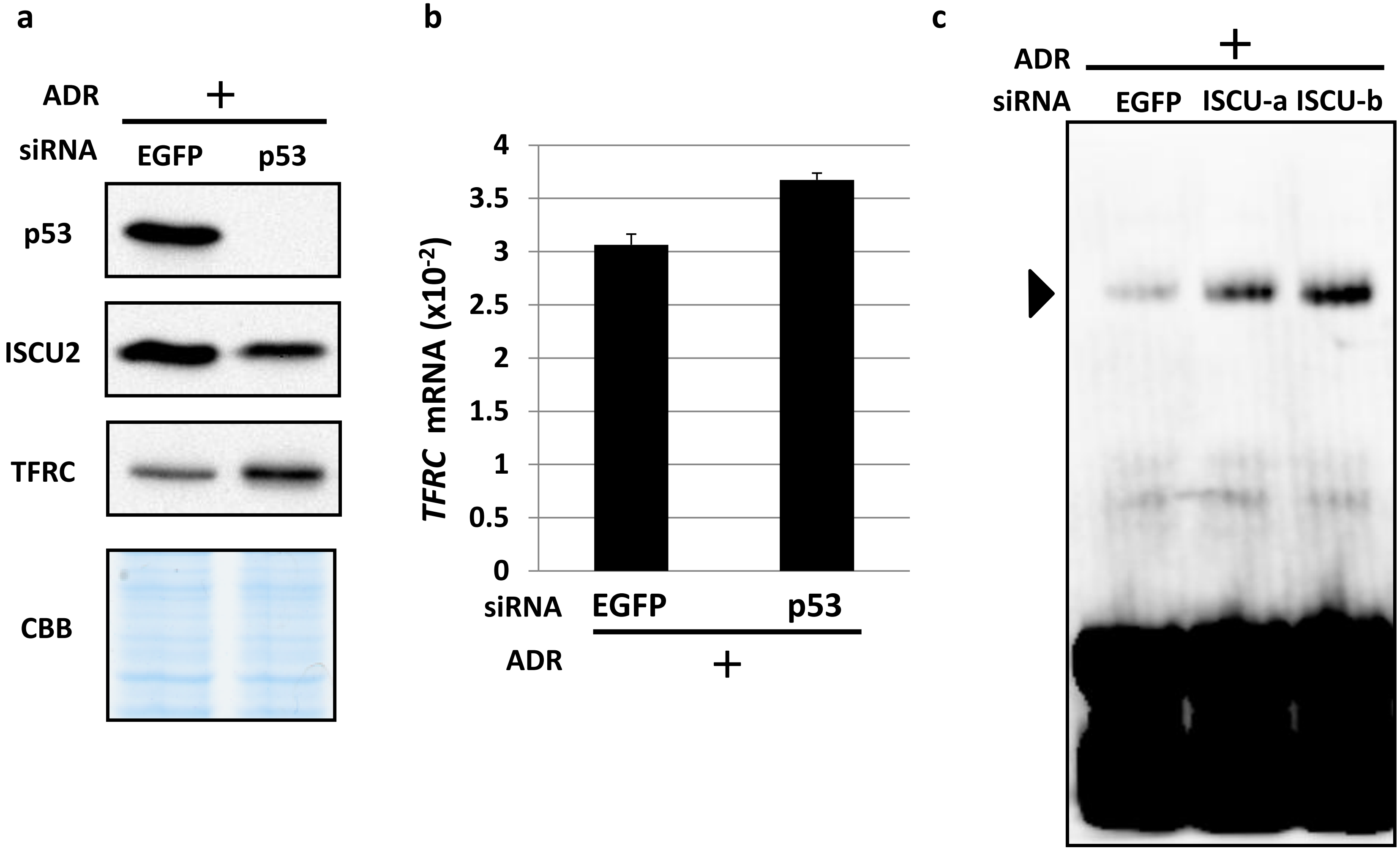

Supplementary Figure 4. Regulation of TFRC by p53-ISCU pathway.

(a) and (b) At 24 h after transfection of each siRNA, HCT116 (p53 wild-type) cells were treated with adriamycin (ADR). At 36 h after treatment, cells were subjected to western blot analysis (a) or qPCR analysis (b). siRNA against EGFP was used as control. CBB staining was shown for loading control (a). *ACTB* was used for the normalization of expression levels (b). Error bars represent S.D. (n = 3). (c) At 24 h after transfection of each siRNA, HCT116 cells were treated with ADR. At 36 h after treatment, cytosolic fractions of cells were incubated with TFRC probe for 30 min and RNA EMSA was performed. Arrowhead indicates protein-RNA complex.

Supplementary Figure 5

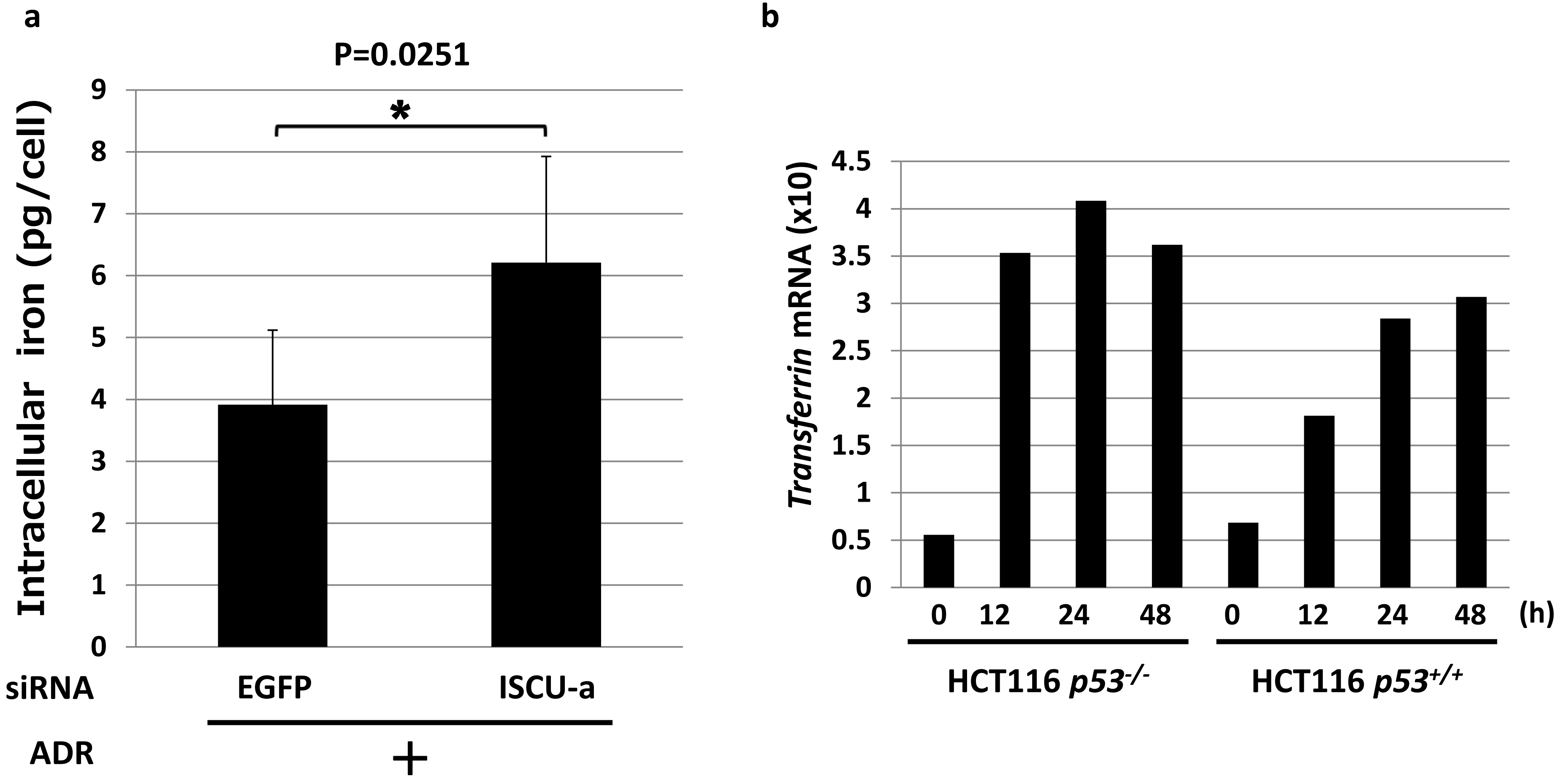

Supplementary Figure 5. ISCU modulated intracellular iron level.
(a) At 24 h after transfection of each siRNA, HCT116 cells were treated with 2ug/ml of Adriamycin (ADR) for 2 h. At 36 h after treatment, cells were collected to measure intracellular iron level. Error bars represent SD (n = 6). *, $P < 0.05$. (b) Expression of *transferrin* mRNA in ADR-treated HCT116 *p53*$^{-/-}$ or HCT116 *p53*$^{+/+}$ cells.

Supplementary Figure 6

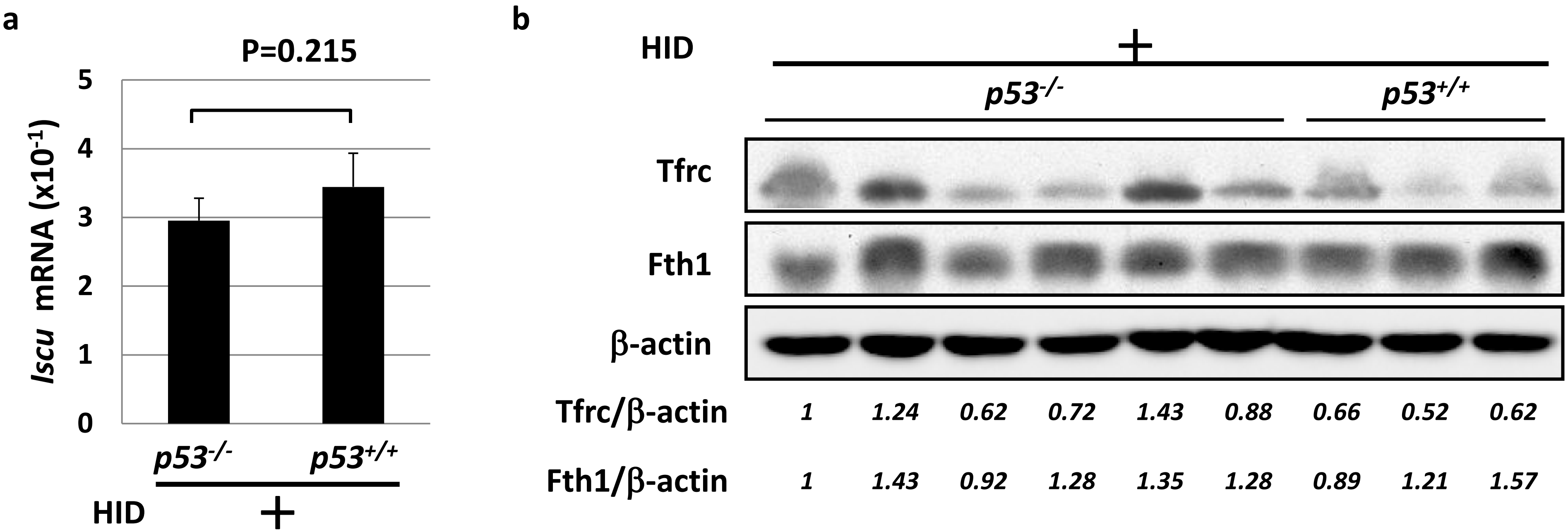

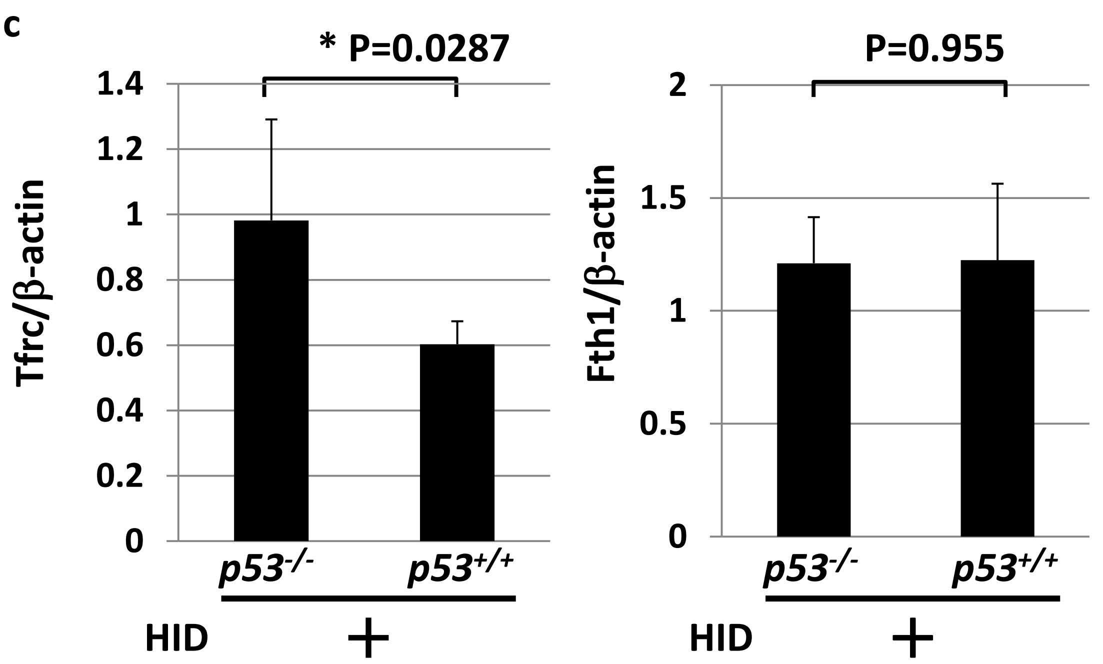

Supplementary Figure 6. Expression of Iscu, Tfrc, and Fth1 in mouse liver tissues after high-iron diet. *p53*$^{+/+}$ (n = 3) or *p53*$^{-/-}$ (n = 6) mice were fed with a high-iron diet (HID) at 6 weeks of age for 3 weeks. At 9 weeks of age, liver tissues were collected for quantitative PCR (qPCR) analysis of *Iscu* mRNA (a) or western blot analysis (b). Tfrc, Fth1, and β-actin expressions from (b) were measured using Image Quant TL Analysis Toolbox (GE healthcare life science, Piscataway, NJ, USA) according to the manufacturer's instructions (c). β-actin was used for the normalization of expression levels. First sample was taken as 1. Error bars represent S.D. *, P<0.05.

Supplementary Figure 7

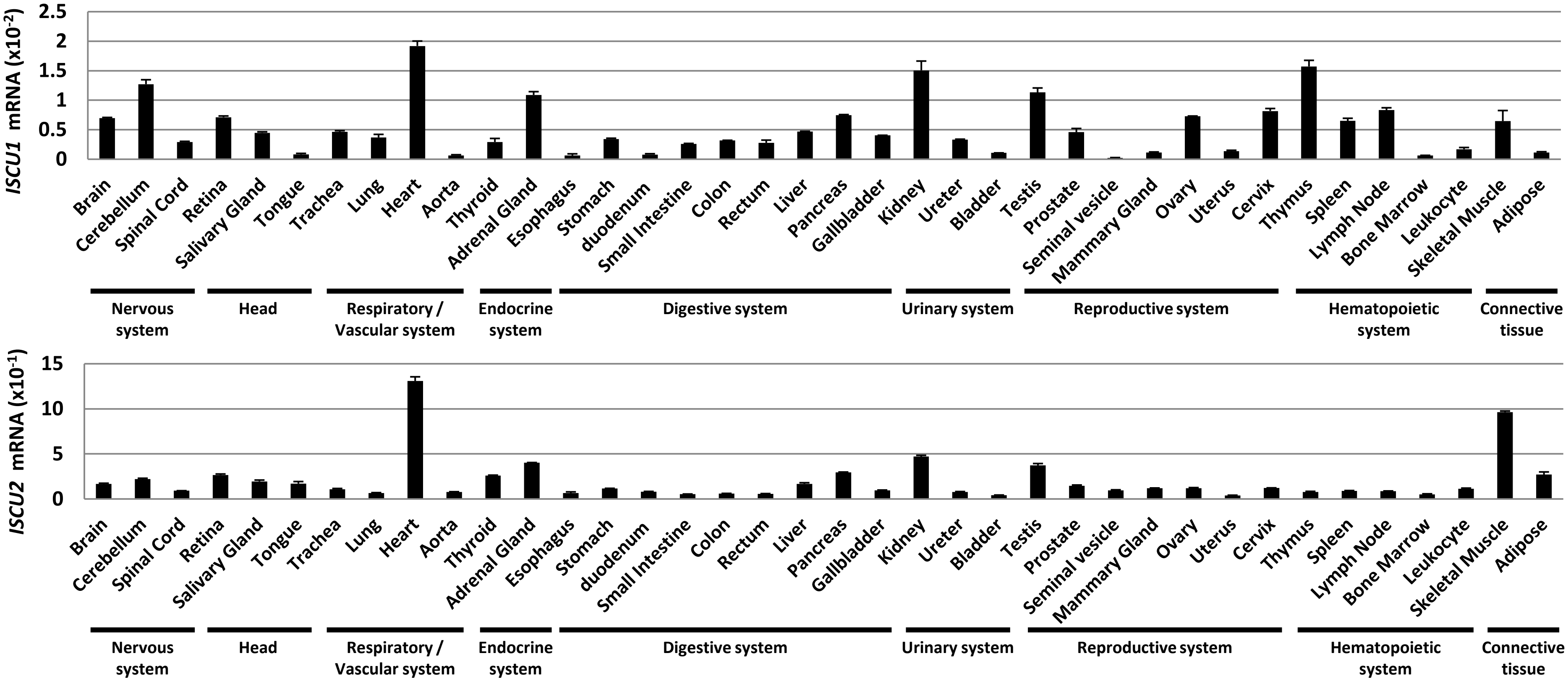

Supplementary Figure 7. Expression of *ISCU* in normal human tissues.
qPCR analysis of *ISCU* in 38 normal human tissues. *ACTB* was used for the normalization of expression levels.

Supplementary Figure 8

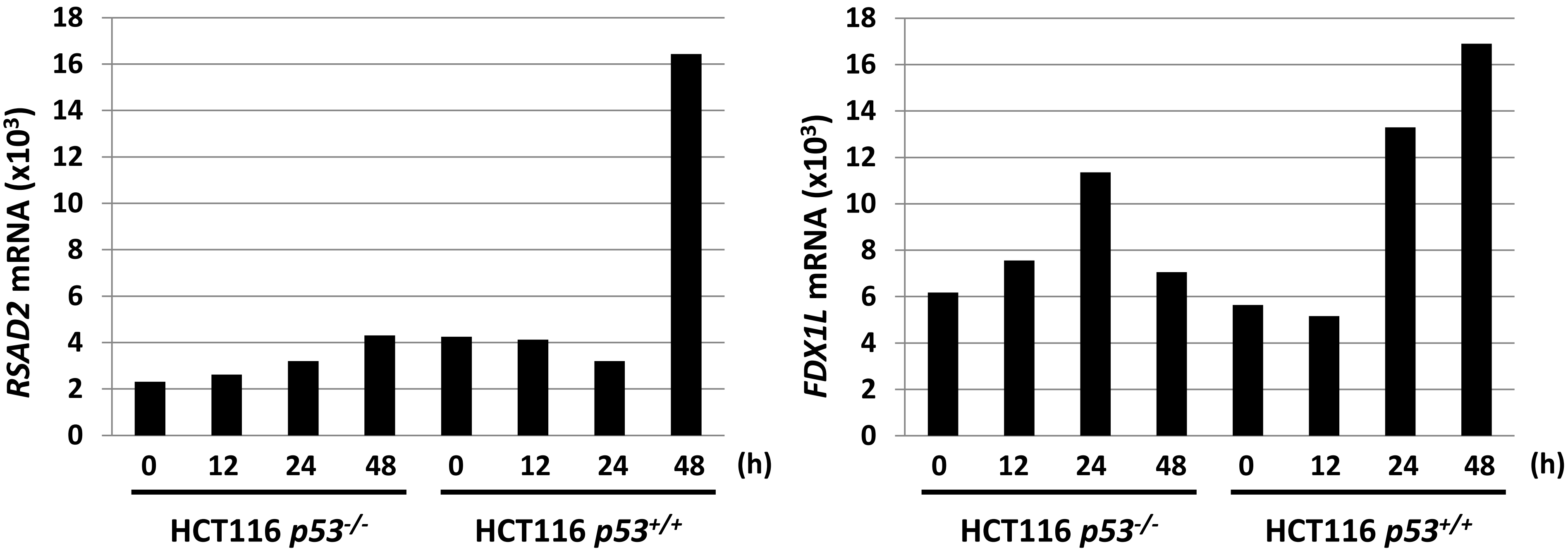

Supplementary Figure 8. The regulation of Fe-S proteins by p53.
Expression of *FDX1L* and *RSAD2* mRNA in ADR-treated HCT116 *p53*$^{-/-}$ or HCT116 *p53*$^{+/+}$ cells.

Supplementary Table 1. Sequences of DNA and RNA oligonucleotides.

Cloning	Forward	Reverse
ISCU1	AAAGAATTCTCACAAATGGTTCTCATTGA	AAACTCGAGGAGGGCTTTCTTCTCTGCCT
ISCU2	AAAGAATTCGGCAAGATGGCGGCGGCTGG	AAACTCGAGGAGGGCTTTCTTCTCTGCCT
mutant ISCU	GATGAAAAGGGGAAAATCGTAGACGCACGCTTCAAAACAT TTGGCTGTGGTTC	CACTTGAATCTGTAACTTCATAACATCCCCGCATGCTGGAG CCCCCACCAGTCCAGT

siRNA oligonucleotides	sense	antisense
siISCU-a	UGUGGUGACGUAAUGAAAUTT	AUUUCAUUACGUCACCACATT
siISCU-b	GAUUGUGGAUGCUAGGUUUTT	AAACCUAGCAUCCACAAUCTT
siEGFP	GCAGCACGACUUCUUCAAGTT	CUUGAAGAAGUCGUGCUGCTT
siISCU1-a	GGUAUCUCAAAUCUGUGAATT	UUCACAGAUUUGAGAUACCTT
siISCU1-b	GUCACAAAUGGUUCUCAUUTT	AAUGAGAACCAUUUGUGACTT
siISCU1-c	GGUUCUCAUUGACAUGAGUTT	ACUCAUGUCAAUGAGAACCTT
siISCU2	UCACAAGAAGGUUGUUGAUTT	AUCAACAACCUUCUUGUGATT
sip53	GACUCCAGUGGUAAUCUACTT	GUAGAUUACCACUGGAGUCTT
siIRP1	GCCAUUGGAUCCUGUACAATT	UUGUACAGGAUCCAAUGGCTT

Quantitative real-time PCR	Forward	Reverse
ISCU	AACACAGATATCGCCAAGGAG	TTTGGGTTCTTGTTTCAATTTGT
ISCU1	TCATTGACATGAGTGTAGACCTTTC	CACCAGTCCAGTTCCAACATT
ISCU2	ACTCTATCACAAGAAGGTTG	CCACAATCTTCCCCTTTTCA
FTH1	GCCAGAACTACCACCAGGAC	CATCATCGCGGTCAAAGTAG
TFRC	CAATGATCGTGTCATGAGAGTG	TAAAGCTGGCAGCGTGTG
ACTB	CCCTGGAGAAGAGCTACGAG	TGAAGGTAGTTTCGTGGATGC
IRP1	GCAGGCACCACAGACTATCC	CAGCAGCATCAAACACATCA
mIscu	CCTGTGAAACTGCACTGCTC	TCTCTGGCTCCTCCTTCTTG
mActb	CTAAGGCCAACCGTGAAAAG	ACCAGAGGCATACAGGGACA
mWaf1	TCCACAGCGATATCCAGACA	GGACATCACCAGGATTGGAC

Gene repoter assay	Forward	Reverse
p53BR	AGGGTCCTGTTGGGACTTGT	CAAAAACATTACGCATGTTGG
p53BRmt	GGATTTTTCTTTCTGAGTCCAGGGACA	CAAAAGAACCCTGCCTCCTGGGCTGTT
mp53BS	ATACAAGCAC	AAGCATACGT

ChIP assay	Forward	Reverse
Genomic fragment including p53BS	CTTCGTGCGTCCCAATTTTA	GTGTGTCTGTCCCTGGACTC

RNA EMSA	seqences
biotinilated IRE from 5' UTR of *FTH1*	UCUUGCUUCAACAGUGUUUGAACGGAAC
biotinilated IRE from 3' UTR of *TFRC*	AAUUAUCGGGAACAGUGUUUCCCAUAAUU

Supplementary Table 2. Association between ISCU-positivity in hepatocellular carcinoma tissues and patients' characteristics (n=92).

		Total<br>n = 92	Strong expression<br>n =45	Weak expression<br>n = 33	Absent expression<br>n = 14	P-value Strong vs Weak and Absent
Gender						
	Male	69	32	26	11	0.4736
	Female	23	13	7	3	
Age (years)						
	< 65	28	16	8	4	0.3667
	65=<	64	29	25	10	
Virus Infection						
	HBV	14	6	8	0	
	HCV	57	27	18	12	0.3135*
	No infection	21	12	7	2	
pT factor						
	T1	66	27	27	12	0.0202**
	T2-T4	26	18	6	2	
p53 staining						
	Positive	26	9	11	6	0.107
	Negative	66	36	22	8	
Ferritin						
	Positive	72	32	26	14	0.1319
	Negative	20	13	7	0	

* HCV and HBV positive vs no infection

**P < 0.05 (Fisher's exact test)
